# Generation of KCL031 clinical grade human embryonic stem cell line

**DOI:** 10.1016/j.scr.2015.12.033

**Published:** 2016-01

**Authors:** Laureen Jacquet, Victoria Wood, Neli Kadeva, Glenda Cornwell, Stefano Codognotto, Carl Hobbs, Emma Stephenson, Dusko Ilic

**Affiliations:** a Stem Cell Laboratories, Division of Women's Health, Faculty of Life Sciences and Medicine, King's College London and Assisted Conception Unit, Guys' Hospital, London, United Kingdom; b Histology Laboratory, Wolfson Centre for Age-Related Diseases, Faculty of Life Sciences and Medicine, King's College London, United Kingdom

## Abstract

The KCL031 human embryonic stem cell line was derived from a normal healthy blastocyst donated for research. The ICM was isolated using laser microsurgery and plated on γ-irradiated human foreskin fibroblasts. Both the derivation and cell line propagation were performed in an animal product-free environment and under current Good Manufacturing Practice (cGMP) standards. Pluripotent state and differentiation potential were confirmed by in vitro and in vivo assays.

## 1 Resource table

**Table:** 

Name of stem cell line	KCL031
Institution	King's College London, London UK
Derivation team	Neli Kadeva, Victoria Wood, Glenda Cornwell, Stefano Codognotto, Emma Stephenson
Contact person and email	Dusko Ilic, email: dusko.ilic@kcl.ac.uk
Date archived/stock date	Aug 02, 2011
Type of resource	Biological reagent: cell line
Sub-type	Human pluripotent stem cell line
Origin	Human embryo
Key marker expression	Pluripotent stem cell markers: NANOG, OCT4, TRA-1-60, TRA-1-81, alkaline phosphatase (AP) activity
Authentication	Identity and purity of line confirmed
Link to related literature (direct URL links and full references)	1) Jacquet, L., Stephenson, E., Collins, R., Patel, H., Trussler, J., Al-Bedaery, R., Renwick, P., Ogilvie, C., Vaughan, R., Ilic, D., 2013. Strategy for the creation of clinical grade hESC line banks that HLA-match a target population. EMBO Mol. Med. 5 (1), 10–17. doi: 10.1002/emmm.201201973 http://www.ncbi.nlm.nih.gov/pubmed/23161805 2) Canham, A., Van Deusen, A., Brison, D.R., De Sousa, P., Downie, J., Devito, L., Hewitt, Z.A., Ilic, D., Kimber, S.J., Moore, H.D., Murray, H., Kunath, T., 2015. The molecular karyotype of 25 clinical-grade human embryonic stem cells lines. Sci. Rep. 5, 17258. doi: 10.1038/srep17258 http://www.ncbi.nlm.nih.gov/pubmed/26607962 3) Ilic, D., Stephenson, E., Wood, V., Jacquet, L., Stevenson, D., Petrova, A., Kadeva, N., Codognotto, S., Patel, H., Semple, M., Cornwell, G., Ogilvie, C., Braude, P., 2012. Derivation and feeder-free propagation of human embryonic stem cells under xeno-free conditions. Cytotherapy. 14 (1), 122–128. doi: 10.3109/14653249.2011.623692 http://www.ncbi.nlm.nih.gov/pubmed/22029654 4) Stephenson, E., Jacquet, L., Miere, C., Wood, V., Kadeva, N., Cornwell, G., Codognotto, S., Dajani, Y., Braude, P., Ilic, D., 2012. Derivation and propagation of human embryonic stem cell lines from frozen embryos in an animal product-free environment. Nat. Protoc. 7 (7), 1366–1381. doi: 10.1038/nprot.2012.080 http://www.ncbi.nlm.nih.gov/pubmed/22722371
Information in public databases	KCL031 is a National Institutes of Health (NIH) registered hESC line NIH Registration Number: NIHhESC-14-0263 http://grants.nih.gov/stem_cells/registry/current.htm?id=672
Ethics	The hESC line KCL031 is derived under license from the UK Human Fertilisation and Embryology Authority (research licence numbers: R0075 and R0133) and also has local ethical approval (UK National Health Service Research Ethics Committee Reference: 06/Q0702/90). Informed consent was obtained from all subjects and the experiments conformed to the principles set out in the WMA Declaration of Helsinki and the NIH Belmont Report. No financial inducements are offered for donation.

## 2 Resource details

**Table:** 

Consent signed	Nov 26, 2008
Embryo thawed	Jun 29, 2011
UK Stem Cell Bank Deposit Approval	Reference: SCSC12-37
Sex	Male 46, XY
Grade	Clinical
Disease status	Healthy/Unaffected
Karyotype (aCGH)	50 kb deletion at 7q22.3 (105,465,968–105,516,305).
SNP Array	Loss at 8q24.23 (136,718,037–136,837,768) (Canham et al., 2015)
DNA fingerprint	Allele sizes (in bp) of 16 microsatellite markers specific for chromosomes 13, 18 and 21 (Jacquet et al., 2013)
HLA typing	HLA-A 02, 24; B 51, 52; Bw 4; C 12, 14; DRB1 11, 15; DRB3 02; DRB5 01; DQB1 03, 06 (Jacquet, L., et al., 2013, Canham, A., et al., 2015)
Viability testing	Pass
Mycoplasma	Negative
Sterility	Pass
Pluripotent markers (immunostaining) (Fig. 1)	NANOG, OCT4, TRA-1-60, TRA-1-81, AP activity
Three germ layer differentiation in vitro (immunostaining) (Fig. 2)	Endoderm: AFP (α-fetoprotein) Ectoderm: TUBB3 (tubulin, beta 3 class III) Mesoderm: ACTA2 (actin, alpha 2, smooth muscle)
Three germ layer differentiation in vivo (teratomas) (Fig. 3)	Endoderm: AFP, GATA4 Ectoderm: TUBB3, GFAP (glial fibrillary acidic protein) Mesoderm: DES (desmin), Alcian Blue and periodic acid–Schiff (PAS)-stained cartilage
Targeted differentiation (Fig. 4)	Cardiomyocytes: TNNT2 (cardiac troponin T) immunostaining
Sibling lines available	No

**Fig. 1:**
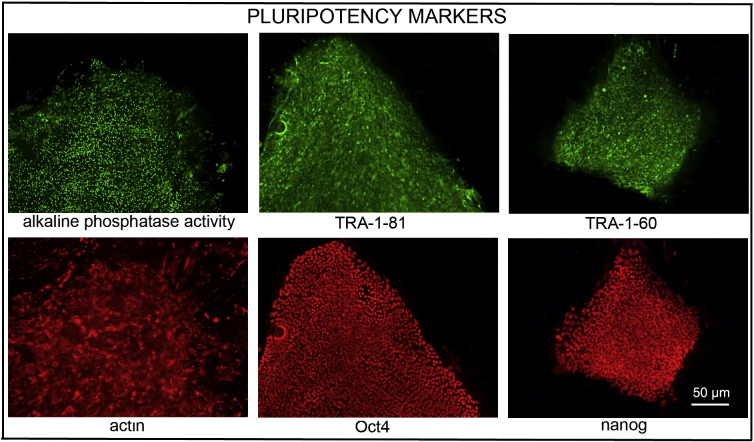
Expression of pluripotency markers. Pluripotency is confirmed by immunostaining (Oct4, Nanog, TRA-1-60, TRA-1-81) and alkaline phosphatase (AP) activity assay. Actin stress fibers, visualized with rhodamine-phalloidin (red), are present in both feeders and hES cell colonies, whereas AP activity (green) is detected only in hES cells. Scale bar, 10 μm.

**Fig. 2:**
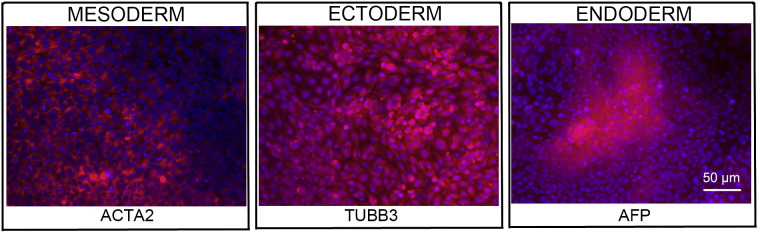
Differentiation of three germ layers in vitro is confirmed by detection of markers: smooth muscle actin (red) for mesoderm, β-III tubulin (red) for ectoderm and α-fetoprotein (red) for endoderm. Nuclei are visualized with Hoechst 33,342 (blue). Scale bar, 50 μm.

**Fig. 3:**
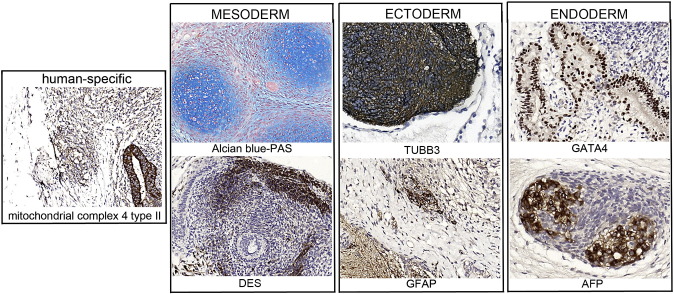
Differentiation of three germ layers in vivo. Teratomas were encapsulated and did not invade surrounding tissue. Sections are counterstained with hematoxylin and eosin and specific stains are brown (immunohistochemistry) or light blue (Alcian blue). Germ layer markers: Alcian blue–PAS-stained cartilage and DES for mesoderm, TUBB3 and GFAP for ectoderm, GATA4 and AFP for endoderm. Positive immunostaining for complex IV type II marker confirms the human origin of the tumor (adjacent section of the one stained for desmin). Scale bars are 100 μm.

**Fig. 4:**
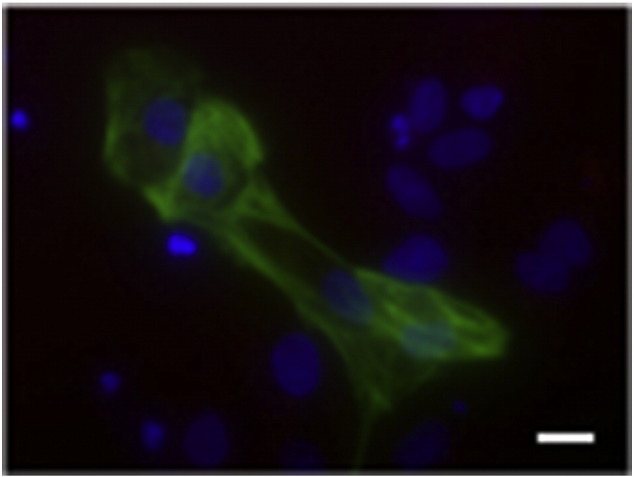
TNNT2 (green) immunostaining on day 30 of cardiac differentiation. Nuclei are visualized with Hoechst 33,342 (blue). Scale bar, 10 μm.

We generated KCL031 clinical grade hESC line following protocols, established previously (Ilic, D., et al., 2012, Stephenson, E., et al., 2012), and now adapted to cGMP conditions. The expression of the pluripotency markers was tested after freeze/thaw cycle (Fig. 1). Differentiation potential into three germ layers was verified in vitro (Fig. 2) and in vivo (Fig. 3), as well as targeted differentiation into cardiac myocytes (Fig. 4).

Molecular karyotyping using array comparative genomic hybridization aCGH identified deletion at 7q22.3 (105,465,968–105,516,305). Whole-genome single nucleotide polymorphism (SNP) array analysis detected loss at 8q24.23 (136,718,037–136,837,768) (Canham et al., 2015). The gain contains no genes and it has been also reported previously to occur in healthy individuals from worldwide population (Macdonald et al., 2014). Estimated frequency in the human population is 3.85% (Canham et al., 2015).

Donors were tested negative for Human Immunodeficiency Virus 1 (HIV1), Hepatitis B (HepB, HCB) and C Virus (HepC, HCV). We did not retest the line.

We also generated research grade of KCL031 line that is adapted to feeder-free conditions.

## 3 Materials and methods

### 3.1 Consenting process

We distribute Patient Information Sheet (PIS) and consent form to the in vitro fertilization (IVF) patients if they opted to donate to research embryos that were stored for 5 or 10 years. They mail signed consent back to us and that might be months after the PIS and consent were mailed to them. If in meantime new versions of PIS/consent are implemented, we do not send these to the patients or ask them to re-sign; the whole process is done with the version that was given them initially. The PIS/consent documents (FRO-V.5) were created on Aug. 10, 2007. HFEA Code of Practice that was in effect at the time of document creation: Edition 7 — R.1 (http://www.hfea.gov.uk/2999.html). The donor couple signed the consent on Nov. 26, 2008. HFEA Code of Practice that was in effect at the time of donor signature: Edition 7 — R.4. HFEA Code of Practice Edition 7 — R.1 was in effect until Dec. 09, 2007, whereas 7 — R.4 was in effect: Oct. 02, 2008–Sep. 30, 2009.

### 3.2 Embryo culture and micromanipulation

Embryo culture and laser-assisted dissection of inner cell mass (ICM) were carried out as previously described in details (Ilic, D., et al., 2012, Stephenson, E., et al., 2012). The cellular area containing the ICM was then washed and transferred to plates containing mitotically inactivated human neonatal foreskin fibroblasts (HFF).

### 3.3 Cell culture

ICM plated on mitotically inactivated HFF were cultured as described (Ilic, D., et al., 2012, Stephenson, E., et al., 2012). TE cells were removed mechanically from outgrowth (Ilic, D., et al., 2007, Ilic, D., et al., 2010). hESC colonies were expanded and cryopreserved at the third passage.

### 3.4 Viability test

Straws with the earliest frozen passage (p.2–3) are thawed and new colonies are counted three days later. These colonies are then expanded up to passage 8, at which point cells were part frozen and part subjected to standard battery of tests (pluripotency markers, in vitro and in vivo differentiation capability, genetics, sterility, mycoplasma).

### 3.5 Pluripotency

Pluripotency in vitro was assessed using two different techniques: enzymatic activity assay [alkaline phosphatase (AP) assay] and immunostaining as described (Ilic, D., et al., 2012, Stephenson, E., et al., 2012).

### 3.6 Differentiation

Spontaneous differentiation into three germ layers was assessed in vitro and in vivo as described (Stephenson, E., et al., 2012, Petrova, A., et al., 2014). Targeted differentiation in cardiomyocytes followed the protocols described earlier (Laflamme, M. A., et al., 2007, Jacquet, L., et al., 2015).

### 3.7 Genotyping

DNA was extracted from hESC cultures using a Chemagen DNA extraction robot according to the manufacturer's instructions. Amplification of polymorphic microsatellite markers was carried out as described (Ilic et al., 2012). Allele sizes were recorded to give a unique fingerprint of each cell line.

### 3.8 Array comparative genomic hybridization (aCGH)

aCGH was performed as described in details (Ilic et al., 2012).

### 3.9 Whole-genome single nucleotide polymorphism (SNP) array

SNP array was performed as described in details (Canham et al., 2015).

### 3.10 HLA typing

HLA-A, -B and -DRB1 typing was performed with a PCR sequence-specific oligonucleotide probe (SSOP; Luminex, Austin, TX, USA) hybridization protocol at the certified Clinical Transplantation Laboratory, Guy's and St Thomas' NHS Foundation Trust and Serco Plc. (GSTS) Pathology (Guy's Hospital, London, UK) as described (Jacquet et al., 2013). HLA typing was also performed independently by other group (Canham et al., 2015).

## 4 Author disclosure statement

There are no competing financial interests in this study.
